# Perspective: Why Organizational Researchers Should Consider Psychophysiology When Investigating Emotion?

**DOI:** 10.3389/fpsyg.2020.01705

**Published:** 2020-07-23

**Authors:** Mathieu Lajante, Gulliver Lux

**Affiliations:** ^1^Department of Marketing, Ted Rogers School of Management, Ryerson University, Toronto, ON, Canada; ^2^Accounting Department, ESG-UQAM, UQAM University, Montreal, QC, Canada

**Keywords:** psychophysiology, emotion, organization science, neuroscience, affective science, organizational behavior, electrophysiology

## Abstract

Although neuroscience is currently gaining wide acceptance in organization science and management studies, some important questions remain unanswered and may hinder the development of so-called organizational neuroscience. Specifically, it appears that the usefulness and the applications of neuroscience methods to organization science are still unclear. Hence, the paper addresses this by examining the role of psychophysiology in investigating implicit emotional experience in organizational behaviors and by discussing how concepts from affective sciences and psychophysiological methods could provide a more complete picture of emotion in organization. Finally, we discuss some important points that should be carefully considered before using psychophysiological measurements in organization science.

## Introduction

The implicit hypothesis of most dominant theories in organization science is that individuals can consciously report their emotions and subsequent attitudes. However, experimental evidence documents that such psychological processes can be more implicit (for a review, see Lindquist et al., 2012). Neuroscientists (e.g., Bechara et al., 2000), behavioral economists (e.g., Kahneman, 2011), and later organizational researchers (e.g., Ashkanazy and Ashton-James, 2005) have pointed out that human consciousness is limited, which should encourage organization science to consider implicit affective processes as well (Barsade et al., 2009). Nevertheless, only few studies have embraced an experimental approach to distinguish implicit from explicit emotional episodes at the workplace. Therefore, we assume that psychophysiology, defined as “the scientific study of social, psychological, and behavioral phenomena as related to and revealed through physiological principles and events in functional organisms” (Cacioppo et al., 2007, p. 4) can improve our understanding of emotion in organizations – this is the viewpoint we present in this perspective paper.

Psychophysiology aims to investigate psychological processes (e.g., emotions) through the observance of physiological correlates (e.g., facial expressions) (Lajante and Ladhari, 2019). The psychophysiological study of emotion would help organizational researchers to explore the role of workplace events in eliciting emotions and how these emotional episodes shape both physiological experience and action tendencies that influence the formation of subsequent individuals’ attitudes and behaviors.

Several psychophysiological methods are convenient for investigating emotion. Peripheral electrophysiological methods, such as facial electromyography (an implicit measurement of emotional valence) and skin conductance responses (an implicit measurement of emotional arousal), or central electrophysiological methods, such as EEG asymmetry (an implicit measurement of emotional engagement and motivational tendencies), are suitable for investigating individuals’ emotions at the workplace and have been extensively used in psychology and marketing. But what about organization science?

There are at least two main explanations for the reticence about using psychophysiology in this field. First, researchers might be unfamiliar with all the psychophysiological methods that are available and how these methods can be applied into organization science. Second, the claim for a psychophysiological approach in organization science relies heavily on brain imaging techniques, which can overshadow other psychophysiological methods.

The main goal of this perspective paper is therefore to discuss how psychophysiology can provide a more complete picture of emotion in organization science. It consists of answering three questions related to implicit emotional episodes in organizational behaviors: What is an emotion? Why do emotions matter in organization? And what do organizational researchers should consider when applying psychophysiology in investigating individuals’ emotion at the workplace?

## What Is an Emotion?

A psychophysiological investigation of emotions in organizations needs a relevant conceptual background to interpret results and discuss inferences. Since any event can elicit a large range of emotion at the workplace (Ashkanazy and Ashton-James, 2005), basic emotion theories – categorical or dimensional – might be too narrow for investigating emotions in organization given their limited set of discrete emotions or dimensions (Scherer and Ellgring, 2007a, b). In contrast, the theories of cognitive appraisal (Moors, 2014) form an appropriate theoretical background for psychophysiological investigations of emotion in organization science. Especially, the Component Process Model (CPM; Scherer, 2009), attaches particular importance to the rapid, ephemeral, and dynamic nature of emotions and considers “the possibility of an infinite number of different types of emotion episode […] driven by the recursively generated appraisal results” (Scherer, 2009, p. 1316).

The CPM relies on a utilitarian approach to emotion in order to predict the determinants triggering and differentiating between distinct emotional episodes (Grandjean and Scherer, 2008). Emotion represents here “a dynamic episode in the life of an organism that involves a process of continuous change in all of its subsystems (e.g., cognition, motivation, physiological reactions, motor expressions, and feelings – the components of emotion) to adapt flexibly to events of high relevance and potentially important consequences” (Grandjean et al., 2008, p. 485). The trigger and the nature of an emotional episode relies on *appraisal*, an ongoing, recursive process of subjective evaluation of an event. Four main criteria outline this appraisal process: (1) relevance – the detection of a significant event in the environment; (2) implication – the potential implications and outcomes of the identified event; (3) coping – the evaluation of the individual’s potential to cope with the event; and (4) normative significance – the appraisal of the interpretation of the adaptive actions by the other members of the reference group (Sander et al., 2005, p. 319). The result of this four criteria-based appraisal process describes the pattern of individual’s physiological and motor expressive reactions, as well as subjective feelings, at the origin of behavioral readiness (i.e., approach/avoidance tendencies). During an emotional episode, physiological changes are correlated to a relevant event and stimulate the body to prepare an adaptive behavioral response. Accordingly, the CPM assumes that the appraisal process of all the four main criteria has a direct influence on the activation of the physiological components of emotion (i.e., autonomous, somatic, and central nervous system activation).

Two important point should be considered here. First, the appraisal criteria are supposed to be processed unconsciously and sequentially, in a fixed order. Moreover, the result of the appraisal process is highly subjective. Several factors such as individual differences, transitional motivational states, cultural values, or pressure exerted by the reference group are taken into consideration along this process. Second, the CPM is not intended to be tested as a whole, at one time. Rather, it provides a consistent analysis framework for the psychophysiological study of emotion in which it is possible to focus only on one stage of cognitive appraisal. Thus, it allows some flexibility in order to fit with the features of the stimuli and the experimental contexts specific to organization science.

Finally, there are at least three main reasons to consider the CPM as a guiding theoretical framework for psychophysiological investigations of emotion in organization science. First, the CPM reminds us that an emotion is a multidimensional, multicomponential, episodic, biologically based pattern of perception (Keltner and Gross, 1999) that needs complementary measurements of changes in the emotional components (namely, autonomic, somatic, expressive, subjective, and motivational components). Therefore, it is necessary to adapt the methodological approach for investigating all the components of an emotion and then obtain the more complete picture of emotions. Second, the CPM makes specific assumptions about the determinants that trigger and differentiate emotional episodes. The same event can thus arouse two different emotions in the same person depending on the context, the objectives pursued, etc. Third, the CPM suggests “a concrete mechanism underlying emotional response patterning, allowing very specific hypotheses (predicting appraisal-driven responses based on functional considerations)” (Grandjean and Scherer, 2008, p. 341). Consequently, the richness of emotion differentiation is accounted for, allowing organizational researchers to model individual differences.

## Why Emotions Matter in Organizations?

Emotion matters in organizational behaviors. According to the Affective Events Theory (AET; Weiss and Cropanzano, 1996), work situations play a role of “emotional primes” – any events that are appraised as conducive or obstructive for reaching personal goals (Ashkanazy and Ashton-James, 2005). Especially, the AET assumes that an individual’s work behavior is determined by the way s/he feels at the moment; an individual’s emotional episode to discrete “affective events” determines subsequent attitudes and behaviors (Ashkanazy and Ashton-James, 2005, p. 224).

Following the introduction of the AET, the “affective revolution” (Barsade et al., 2003) has led organization researchers to admit that one of the main sources of emotional episodes originates from individuals’ social and physical environment. An emotional episode “can arise from people’s own internal prior experiences or history, particularly as they interact with physical or interpersonal “primes” they encounter in specific organizational settings” (Barsade et al., 2009, p. 146). Ashkanazy and Ashton-James (2005), p. 225) pointed out that “since an individual’s goals are subjective and situation-dependent, virtually any workplace event can elicit emotional episodes, so long as it is perceived by employees to affect their ability to achieve their personal goals, whatever they may be” – which is in accordance with the definition of emotion in the CPM (Scherer, 2009).

The “affective revolution” (Barsade et al., 2003) also led organization researchers to admit that emotions can take place “at every level of organizational functioning, extending all the way from the top of the organization to the most fundamental level – the day-to-day experiences of organizational members” Ashkanasy(2003, p. 42). Emotional episodes at level 1 (i.e., within-person) have a particular importance compared to the subsequent organizational levels – between-person, interpersonal interactions, groups, and organization-wide – where emotion takes place. At level 1 (i.e., within-person), “neural structures receive and interpret affective stimuli in the workplace environment, and initiate physiological changes that underlie the experience and expression of affect and emotion in the workplace” (Ashkanazy and Ashton-James, 2005, p. 231). Subsequently, emotional episodes influence individuals’ judgments, behavioral motivation (e.g., approach or withdraw), and actions in the organization. Specifically, “emotions that individuals feel at Level 1 are promulgated through the other levels of the model, including the way that organizational members interact with each other and the means by which emotion spread though groups, ultimately affecting the culture and climate of the organization as a whole” (Ashkanazy and Ashton-James, 2005, p. 253).

There is now an extensive literature about the role of individuals’ emotions in organizations (Rafaeli et al., 2009). However, experiments that examine the causes and consequences of emotional episodes at level 1 with psychophysiological measures are scarce, which suggests a methodological myopia. The only use of self-report methods is risky since an emotional episode cannot be reduced to its conscious and verbalized aspect (Russell and Barrett, 1999) – it would just highlight the tip of the iceberg. Ashkanasy (2003) has already stressed the challenge of emotion measurement in organizational science. In his review, he noticed that “one of the reasons that organization science has been so belated in researching emotions is because of the inherently ephemeral and idiosyncratic nature of emotion” (p. 10). Hence, a multimethod approach combining psychophysiological methods with behavioral and self-report methods for investigating emotions as well as implicit psychological measures is central to succeeding in the study of emotion in organization.

For instance, the approach we propose could benefit human resources research dealing with employees’ mood and its impact on performance (Tsai et al., 2007; Chi et al., 2015). Indeed, in these studies, emotion is used as a “marker” of mood (George and Zhou, 2007). Our approach could also benefit to auditing research dealing with the variation in auditors’ emotional states (Guénin-Paracini et al., 2014), as well as studies in the field of accounting, which underlines the importance of emotions among healthcare professionals to explain the relationship to quantification (Amslem and Gendron, 2019). Finally, the approach we propose could also benefit studies in control, which focuses on the role of affective processes during the appropriation process of control tools (Lux and Lajante, 2017; Lajante and Lux, 2018).

## What Do Organizational Researchers Should Consider When Applying Psychophysiology?

**FIGURE 1:**
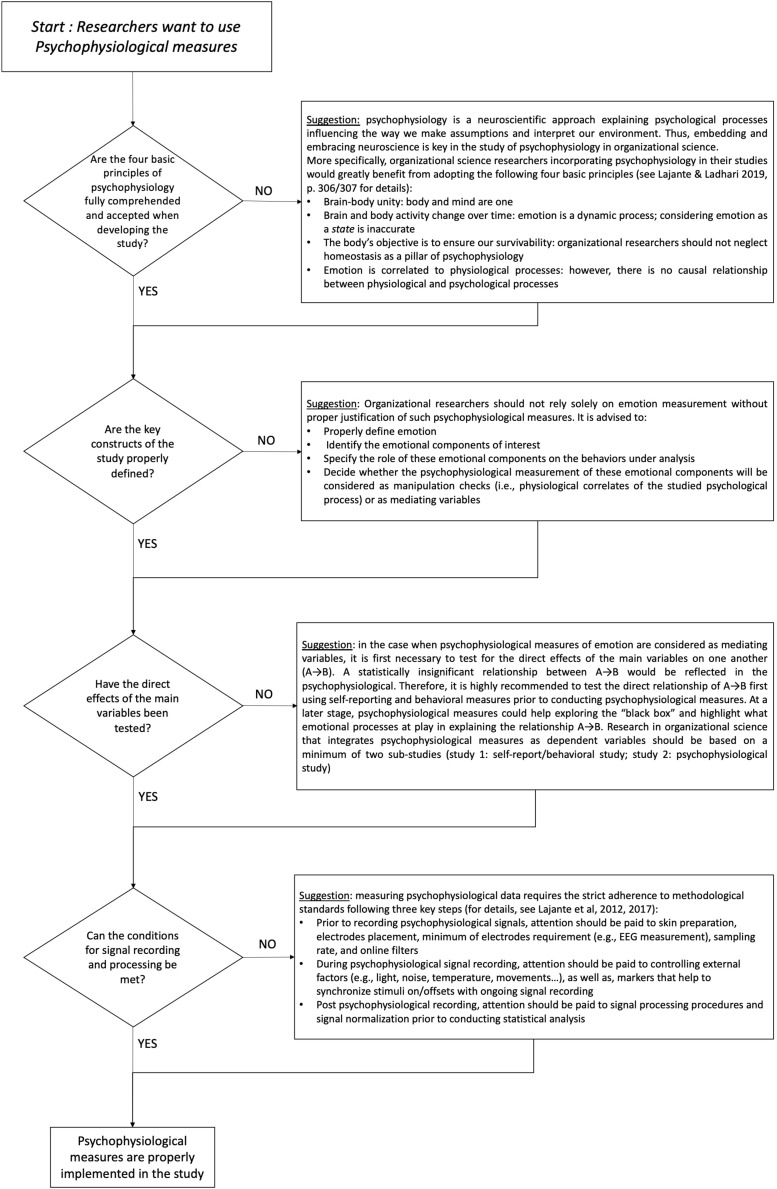
The four sequential conditions to design and implement psychophysiological studies of emotion in organization science. These four sequential conditions work as a checklist to help organizational researchers to evaluate the appropriateness of carrying out a psychophysiological study of emotion, to verify the feasibility of such study, and to anticipate common mistakes and misconceptions when applying psychophysiological measurements in organization science.

A psychophysiological perspective can provide organizational researchers with new opportunities to conceptualize and to investigate previously invisible emotional drivers of organizational behavior (Ashkanasy et al., 2014). In this way, psychophysiology aims to understand the nature of emotion and to disentangle the relationship between emotional episodes and subsequent attitudinal and behavioral responses tailored to the workplace by means of a multimethod approach (Becker et al., 2011; Ashkanasy et al., 2014). However, we think that organizational researchers should follow four sequential conditions to design and implement psychophysiological studies of emotion (Figure 1).

Moreover, five points should be carefully considered when adopting psychophysiological investigations of emotion in organization science.

### Avoiding the “So What?” Issue

Psychophysiological methods are not always appropriate to investigate all affective-related phenomenon in organization science. The method should be selected in accordance with the research question (the “*so what?*” question) as follows: (1) establishing the research question, (2) determining the research objectives, (3) specifying the research design, and then (4) selecting the appropriate methodology. For instance, if the research question relates to the role of emotion in job satisfaction, it would be necessary to define what an emotion is and to determine which component should be measured to answer. As we pointed out, the multicomponent nature of emotion calls for the use of different measures to grasp emotional episodes as comprehensively as possible (Russell and Barrett, 1999). Moreover, different measures are not interchangeable but complementary to the extent that they do not tap the same underlying components of emotions. Finally, no single measurement tool can be considered “the Holy Grail.” Therefore, clearly stating the research question would help to define the underlying process of interest and then justify the use of psychophysiology (e.g., if the studied process is non-observable and/or implicit) by avoiding any arguments of authority such as “the brain is the unique, objective source of information.”

### Embracing the Whole Spectrum of Psychophysiology

Brain imaging techniques are in the frontline of popular expectations when referring to psychophysiology in organization science (e.g., Becker and Cropanzano, 2010; Becker et al., 2011; Ashkanasy et al., 2014; Lindebaum and Jordan, 2014). However, this narrow understanding of psychophysiology does not catch all the complexities of human cognition. First, it is erroneous to reduce psychophysiology to a “toolbox.” Rather, psychophysiology provides meaningful theories that could help to understand the antecedents and the consequences of emotions in organizations. Second, it is erroneous to reduce psychophysiology to the brain (and brain imaging) only. Psychophysiology relies on physiological symptoms related to the whole nervous system – the central nervous system (i.e., the brain, the cerebellum, and the spinal cord) and the peripheral nervous system (i.e., the somatic nervous system and the autonomic nervous system). Therefore, brain activation alone cannot teach us everything about the (emotional) meaning of specific response patterns (especially when they are not conscious). In addition to central nervous system measurements, investigating peripheral responses by means of electrophysiological measurements, such as facial electromyography or skin conductance responses, would also provide sensitive indicators for investigating emotional processes (Cacioppo et al., 2007).

### Paying Attention to Measurement Reliability and Validity

Measurement reliability and validity and confidence in the results rely on compliance with methodological standards when recording, processing, and quantifying physiological signals. Such methodological considerations might sound overly technical and beyond what organizational researchers could digest, but it is a prerequisite for the development of psychophysiological methods in organization science: first, because it provides a guarantee of transparency regarding reliability and validity for those who suspect the psychophysiological approach of being obscure, inefficient, and unreliable; second, because it fosters replication and extension studies. The link between methodological rigor and the spread of psychophysiology has been highlighted by Wiles and Cornwell(1991, pp. 266–267) who stated that “progress toward developing valid physiological measures is thwarted when questionable procedures are implemented. […] A clearer understanding and acceptance of psychophysiological techniques will only come about as evidence of validity and reliability is documented by investigators.” For instance, critical reviews of psychophysiological studies in marketing showed to what extent the methodological bias in electrophysiological signal recording and analyses could have negatively affected results and so the confidence in their possible interpretations (Lajante et al., 2012, 2017).

### Distinguishing Utilitarian From Aesthetic Emotions

In the CPM, an event is sequentially appraised through four main criteria. However, this expensive appraisal process and the subsequent behavioral readiness occur “only if the event concerns a goal or need of importance or when a salient discrepancy with an expected state is detected” (Scherer, 2009, p. 1318). This sequence assumption leads to distinguishing between aesthetic and utilitarian emotions. If the appraisal process is complete (i.e., all the four main criteria have been appraised), then the emotional episode will result in action tendencies. In that case, the emotion is utilitarian “in the sense of having major functions in the adaptation and adjustment of individuals to events that have important consequences for their well-being” (Scherer, 2004). However, if the event appraisal is incomplete, because it is considered as irrelevant for the individual’s goals and needs, then the emotional episode will result in attitudes rather than action tendencies. In that case, the emotional episode is aesthetic because it is “triggered in situations that usually have no obvious material effect on the individual’s well-being and only rarely lead to specific goal-oriented responses” (Scherer and Zentner, 2008, p. 596).

### Distinguishing Conscious and Unconscious Emotional Experiences

Finally, it is important to not directly compare conscious and unconscious emotional experiences. Subjective feelings, which correspond to the conscious part of an emotional episode, constitute one of the components of emotion – just as physiological and expressive components – and have a special status as it integrates and regulates the component processes. Specifically, Grandjean et al.(2008, p. 486) have suggested that “subjective experience serves as a monitoring function, integrating all information about the continuous patterns of change in all other components, as well as their coherence, and then building an integrative conscious representation. Thus, feeling is an extraordinarily complex conglomerate of information from different systems.” Self-report measures and subjective feelings ratings could be easily implemented in a psychophysiological investigation of emotion. It provides information about individuals’ subjective emotional experiences that cannot be reached with other psychophysiological methods and reflect “an emergent propriety of the synchronization of the different subcomponents of an emotional episode” (Scherer, 2004, p. 493). Hence, and even though self-report methods are not “neuroscientific” *per se*, studies aiming to give a complete picture of emotions in organization should always incorporate subjective feelings ratings – and avoid the trap to compare self-report measures of emotion with the psychophysiological ones.

## Conclusion

Although neuroscience is currently gaining wide acceptance in organization science, some important questions remain unanswered and may hinder the development of so-called organizational neuroscience. Specifically, it appears that the usefulness and the applications of psychophysiology to organization science are still unclear. Hence, this perspective paper addresses this by reminding the role of emotion in organizations and by discussing how psychophysiology could provide a more complete picture of emotion. We think that the perspective we provide on the component process model of emotion and the central role of emotion in organizational behavior – its dynamic, implicit, and multicomponential nature – could overcome organization researchers’ reluctance to implement psychophysiological methods, and we are convinced that future results will contribute to enhancing knowledge about emotions in organization science. Finally, we are convinced that our five-point discussion about what should be carefully considered before using psychophysiology in organization science will stimulate researchers to consider the broad array of methods at their disposal to investigate causes and consequences of individuals’ emotional episode at the workplace.

## Data Availability Statement

The original contributions presented in the study are included in the article/supplementary material, further inquiries can be directed to the corresponding author.

## Author Contributions

Both authors listed have made a substantial, direct and intellectual contribution to the work, and approved it for publication. Both authors contributed equally and are listed in alphabetic order.

## Conflict of Interest

The authors declare that the research was conducted in the absence of any commercial or financial relationships that could be construed as a potential conflict of interest.
